# Breakage Ratio of Silicon Wafer during Fixed Diamond Wire Sawing

**DOI:** 10.3390/mi13111895

**Published:** 2022-11-02

**Authors:** Tengyun Liu, Yancai Su, Peiqi Ge

**Affiliations:** 1 Faculty of Mechanical Engineering, Qilu University of Technology (Shandong Academy of Sciences), Jinan 250000, China; 2 School of Mechanical Engineering, Shandong University, Jinan 250000, China; 3 Key Laboratory of High-Efficiency and Clean Mechanical Manufacture at Shandong University, Ministry of Education, Jinan 250061, China

**Keywords:** fixed diamond wire saw, silicon wafer, breakage ratio, forced vibration

## Abstract

Monocrystalline silicon is an important material for processing electronic and photovoltaic devices. The fixed diamond wire sawing technology is the first key technology for monocrystalline silicon wafer processing. A systematic study of the relationship between the fracture strength, stress and breakage rate is the basis for thinning silicon wafers. The external vibration excitation of sawing machine and diamond wire lead to the transverse vibration and longitudinal vibration for silicon wafers. The transverse vibration is the main reason of wafer breakage. In this paper, a mathematical model for calculating breakage ratio of silicon wafer is established. The maximum stress and breakage ratio for as-sawn silicon wafers are studied. It is found that the maximum amplitude of the silicon wafers with the size of 156 mm × 156 mm × 0.2 mm was 160 μm during the diamond wire sawing process. The amplitude, maximum stress and breakage rate of the wafers increased with the increase of the cutting depth. The smaller the silicon wafer thickness, the larger of silicon wafer breakage ratio. In the sawing stage, the breakage ratio of the 156 mm × 156 mm section with a thickness of 0.15 mm of silicon wafers is 6%.

## 1. Introduction

In the photovoltaic industry, wire sawing technology is the first process of silicon wafer processing. Its processing quality and processing cost affect the total manufacturing cost of the photovoltaic industry [1,2,3,4,5]. At present, more than 95% of silicon wafers are cut by diamond wire saws, and its processing cost accounts for about 15% of the total cost of the photovoltaic industry [6,7,8]. Reducing the thickness of silicon wafer and increasing the yield of silicon wafer are measures to reduce the processing cost [9,10,11,12]. Liu et al. [13] calculated that decreasing wafer thickness from 160 to 50 µm can potentially get a capex reduction of USD 0.14/(W/year) and a cost reduction of USD 0.07/W of the current PV module, which in 2019 had an average price of USD 0.26/W [8].

However, with the decrease of silicon wafer thickness and the development of wire sawing technology towards high-speed direction, the breakage ratio of silicon wafer in wire sawing will increase [14,15,16,17]. Therefore, it is necessary to study the influence of silicon wafer thickness and external factors on the mechanical properties of silicon wafer in the sawing process. The research results of some scholars show that the smaller the wafer thickness of monocrystalline silicon, the worse the mechanical stability, and the easier the crack propagation in the wafer, leading to the increase of wafer breakage ratio [18,19,20,21,22].

The fracture strength and stress state of silicon wafers affect the breakage ratio of wafers [23]. A systematic study of the relationship between the fracture strength, stress and breakage ratio is the basis for determining the thickness of wafers [24,25]. In the cutting process of monocrystalline silicon wafers by using resin bonded diamond wire saw, the wafer stress is related to the wafer vibration station. The wafer vibration state is constantly changing, so the wafer stress is constantly changing too. The vibration of monocrystalline silicon wafer in the sawing process includes transverse vibration and longitudinal vibration, and the transverse vibration is the main reason of wafer breakage [26]. Therefore, this chapter mainly studies the transverse vibration of slice and analyzes the maximum stress of slice caused by transverse vibration. The relationship between fracture strength, stress and fragmentation rate was established to determine the slice thickness under a certain fragmentation rate. From now on, there is no relevant report on the research of breakage ratio of silicon wafer during diamond wire sawing.

Hence, a theoretical model is established in order to solve the above problems. The free vibration and forced vibration of silicon wafers are studied based on the theory of elastic thin plate. Breakage ratio of wafers is analyzed by using the Weibull distribution theory. The research results are of great significance for reducing the thickness and breakage rate of silicon wafers and increasing the yield of silicon wafers.

## 2. Model of Wafer Breakage during Diamond Wire Sawing

### 2.1. Free Vibration of Wafer during Wire Sawing

**Figure 1:**
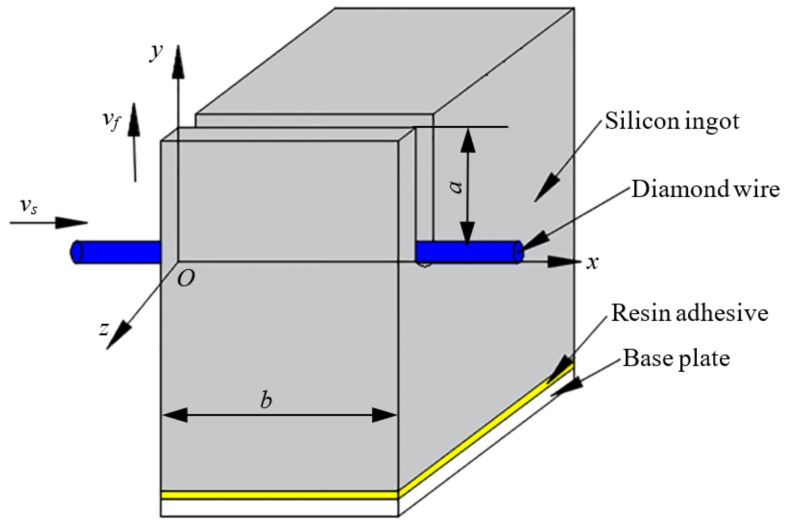
The process of diamond wire sawing.

During fixed diamond wire sawing, the wire periodically moves back and forth to cut the silicon ingot. Diamond abrasives scratch the material and generate cutting force on the silicon wafer. Cutting force, the flow of cutting fluid and machine vibration all have effect on the wafer vibration. The silicon ingot is adhered on the connection plate by using the resin glue. The lateral vibration and tangential vibration are generated under the influence of external excitation. In these two vibrations, the lateral vibration is considered as the major reason of causing wafer break because of a larger stress is generated in the lateral vibration. The thickness of as-sawn silicon wafer for photovoltaic industry is 0.2 mm, which is far less than the length 156 mm. Furthermore, one side of silicon wafer is a fixed side, and the other side is free. Thus, the silicon wafer can be seen as a cantilever plate. Therefore, the silicon wafer is treated as the elastic cantilever plate in this paper. The process of diamond wire sawing silicon ingot is shown in Figure 1.

One coordinate system is established for diamond wire sawing. The origin point is located as the entry point of diamond wire. The axial direction of diamond wire is the *x* direction, and the opposite direction of feed direction is the y direction, as shown in Figure 1. The length of silicon wafer is 156 mm, and this parameter is denoted as *b*. The cutting depth of silicon ingot *a* is from 0~156 mm. The thickness variation of wafers is ignored to simplified calculation model.

The vibration equation for the sliced silicon wafer is obtained based on the thin plate theory:

(1) $$D\nabla^{4}w+\rho h\frac{\partial^{2}w}{\partial t^{2}}=0$$

where *w* is the amplitude of sliced silicon wafer; *D* is the flexural rigidity of wafer, which is related to the elastic modulus *E*, wafer thickness *h* and Poisson’s ratio *μ*, $D=\frac{Eh^{3}}{12(1-\mu^{2})}$. *Ρ* is the density of silicon.

The boundary conditions for cantilever plate are:

(1) fixed boundary (geometric boundary condition
  (2) $${\left(w\right)}_{y=0}=0$$
  (3) $${\left(\frac{\partial w}{\partial y}\right)}_{y=0}=0$$
(2) free boundary
  (4) $${\left(\frac{\partial^{3}w}{\partial x^{3}}+(2-\mu )\frac{\partial^{3}w}{\partial x\partial y^{2}}\right)}_{x=0,x=b.y=a}=0$$
  (5) $${\left(\frac{\partial^{2}w}{\partial y^{2}}+\mu \frac{\partial^{2}w}{\partial x^{2}}\right)}_{x=0,x=b.y=a}=0$$

The method of separation of variables is used to solve the Equation (1) based on the thin plate theory. According to this method, the vibration equation can be written as:

(6) $$w(x,y,t)=W(x,y)\sin(\omega t+\phi_{0})$$

where the *W* (*x,y*) is the shape function; *ω* is the natural frequency of vibration; *φ*_0_ is the initial phase.

The integral-transform method is used to simplify the fourth-order differential equation in order to obtain the shape function. This method transforms the high-order differential equation to a simple system of linear equations. The solution of Equation (1) can be obtained by solving the linear equations with the integral-transform method. The shape function *W* (*x,y*) after transformation is:

(7) $$\begin{array}{l} W(x,y)=\frac{2}{ab}\sum_{m}^{\infty }C_{m0}\left[\frac{q_{m0}}{D}+R_{m0}I_{m}-R_{m0}J_{m}+{\left(-1\right)}^{\frac{m-1}{2}}T_{m0}K_{0}-\frac{\alpha_{m}}{2}L_{0}\right]\sin\frac{\alpha_{m}}{2}x\\ +\frac{4}{ab}\sum_{m}^{\infty }\sum_{n}^{\infty }C_{mn}\left[\frac{q_{m0}}{D}+{\left(-1\right)}^{n}R_{mn}I_{m}-R_{mn}J_{m}+{\left(-1\right)}^{\frac{m-1}{2}}T_{mn}K_{n}-\frac{\alpha_{m}}{2}L_{n}\right]\sin\frac{\alpha_{m}}{2}x\cos\beta_{n}y \end{array}$$

In Equation (7), the coefficients *C_mn_*, *R_mn_*, *T_mn_*, *α_m_*, and *β_n_* can be obtained separately by the following formulas:

$$\begin{array}{c} C_{mn}=\frac{1}{{\left(\alpha_{m}^{2}/4+\beta_{n}^{2}\right)}^{2}-\rho h\omega_{mn}^{2}/D},\;R_{mn}=\nu \frac{\alpha_{m}^{2}}{4}+\beta_{n}^{2},\;T_{mn}=\frac{\alpha_{m}^{2}}{4}+\nu \beta_{n}^{2}\\ \alpha_{m}=\frac{m\pi }{a},\;\beta_{n}=\frac{n\pi }{b}\;(m=1,3,5\ldots ;\;n=0,1,2\ldots ) \end{array}$$

Combining the shape function (7) and the boundary condition Equations (2)–(5), the following equations can be obtained:

(8) $$\begin{array}{l} \sum_{m=1,3}^{\infty }{\left(-1\right)}^{n}\alpha_{m}C_{mn}R_{mn}I_{m}-\sum_{m=1,3}^{\infty }\alpha_{m}C_{mn}R_{mn}J_{m}\\ +\sum_{m=1,3}^{\infty }{\left(-1\right)}^{\frac{m-1}{2}}\alpha_{m}C_{mn}T_{mn}K_{n}-\frac{1}{2}\sum_{m=1,3}^{\infty }\alpha_{m}^{2}C_{mn}L_{n}=0 \end{array}\left(n=0,1,2,\ldots \right)$$

(9) $$\begin{array}{l} \sum_{m=1,3}^{\infty }{\left(-1\right)}^{n+\frac{m-1}{2}}(\nu -C_{mn}R_{mn}T_{mn})I_{m}-\sum_{m=1,3}^{\infty }{\left(-1\right)}^{\frac{m-1}{2}}(\nu -C_{mn}R_{mn}T_{mn})J_{m}\\ +\sum_{m=1,3}^{\infty }{\left(-1\right)}^{m-1}(1-C_{mn}T_{mn}^{2})K_{n}+\frac{1}{2}\sum_{m=1,3}^{\infty }{\left(-1\right)}^{\frac{m-1}{2}}\alpha_{m}^{}C_{mn}T_{mn}L_{n}=0 \end{array}\left(n=0,1,2,\ldots \right)$$

(10) $$\begin{array}{l} \left[1-\nu^{2}+2\sum_{n=1}^{\infty }{\left(-1\right)}^{n}(1-C_{mn}R_{mn}^{2})\right]I_{m}-\left[1-\nu^{2}+2\sum_{n=1}^{\infty }\left(1-C_{mn}R_{mn}^{2}\right)\right]J_{m}\\ +2\sum_{n=1}^{\infty }{\left(-1\right)}^{\frac{m-1}{2}}(\nu -C_{mn}R_{mn}T_{mn})K_{n}+\frac{2\nu }{\alpha_{m}}L_{0}+\sum_{n=1}^{\infty }\alpha_{m}C_{mn}R_{mn}L_{n}=0 \end{array}\left(m=1,3\ldots \right)$$

(11) $$\begin{array}{l} \left[1-\nu^{2}+2\sum_{n=1}^{\infty }\left(1-C_{mn}R_{mn}^{2}\right)\right]I_{m}-\left[1-\nu^{2}+2\sum_{n=1}^{\infty }{\left(-1\right)}^{n}(1-C_{mn}R_{mn}^{2})\right]J_{m}\\ +2\sum_{n=1}^{\infty }{\left(-1\right)}^{n+\frac{m-1}{2}}(\nu -C_{mn}R_{mn}T_{mn})K_{n}+\frac{2\nu }{\alpha_{m}}L_{0}+\sum_{n=1}^{\infty }{\left(-1\right)}^{n}\alpha_{m}C_{mn}R_{mn}L_{n}=0 \end{array}$$

The unknown variables in Equation (11) are *I_m_*, *J_m_*, *K_n_* and *L_n_*. The condition of Equation (11) has non-zero solution is that the matrix determinant describing the coefficient of the system of linear equations is equal to zero. The natural frequency of silicon wafer is obtained by setting the matrix determinant equal to zero. After that, the four variables can be solved. The four variables *I_m_*, *J_m_*, *K_n_*, *L_n_* are the coefficients about four sides of half sine series and bending moment of cosine series according to thin plate theory. Thus, setting *I_m_* = −*J_m_* and *I_m_* = *J_m_* can yield the symmetrical mode and anti-symmetrical mode respectively. Therefore, the Equations (6) and (7) can be known.

### 2.2. Forced Vibration of Wafer during Wire Sawing

(1) Force analysis for silicon wafer

**Figure 2:**
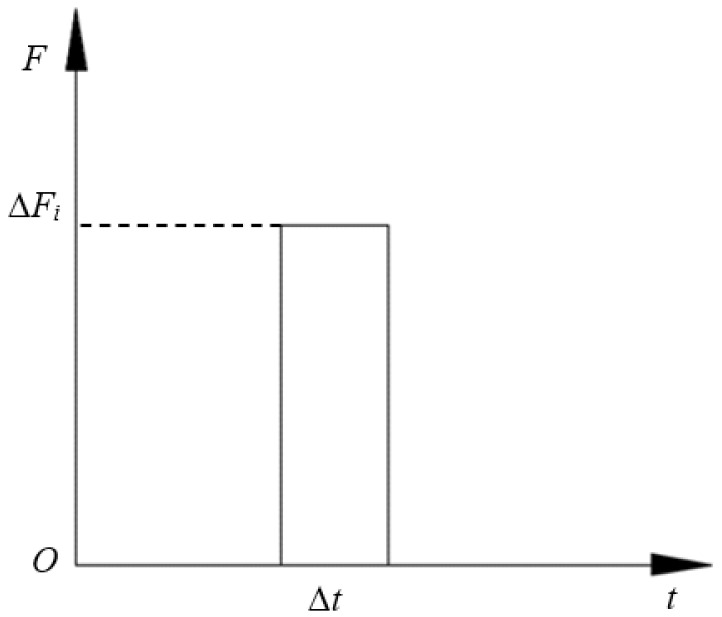
Impulse excitation induced by single grain.

During diamond wire sawing, diamond abrasives scratch on silicon ingot under the drive of wire movement. Each grain generates impact force on the wafer within a certain time. Force induced by each grain can be seen as an impact force which value is ∆*F* and action time is ∆*t*. Thus, the total cutting force is equal to the sum of each action force induced by every grain. The impact force is assumed as an impulse excitation as shown in Figure 2, which value is ∆*F* and action time is ∆*t*. The action time is concerned with the wire speed vs. silicon ingot length *L*, that is:

(12) $$\Delta t=L/v_{s}$$

A lot of diamond abrasives take part in cutting, that means the total cutting force is equal to the sum of each value of impulse excitation at a moment. Because of the shape, diameter and protrusion height for each diamond grain are all different, the cutting depth is various. Hence, the total cutting force changes with shift of time. When the sawing process reaches a steady state, the sawing force is a stationary random fluctuation external excitation. To simplify the cutting process, the total cutting force is simplified as a harmonic force.

(13) $$P(t)=F\sin\omega_{f}t$$

which *ω_f_* is the frequency of cutting force.

**Figure 3:**
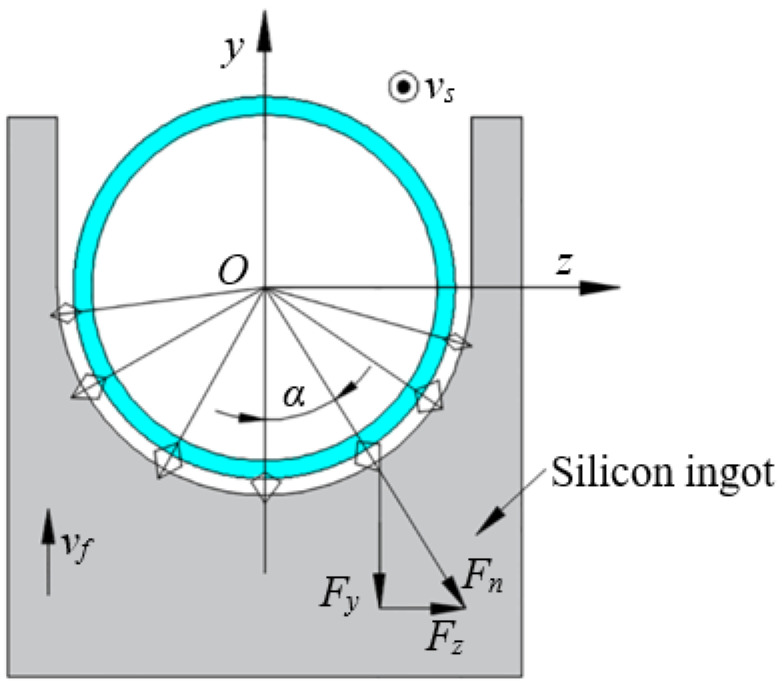
Force analysis for silicon wafer.

Normal cutting force and tangential force are generated during diamond wire sawing. The normal force distributes along the axial direction of diamond wire, which has no effect on the lateral vibration of silicon wafer and not be considered. Hence, the normal force is vertical to the surface of kerf, and this force can be decomposed into two kinds of force, perpendicular silicon wafer surface *F_z_* and parallel to wafer surface respectively *F_y_*, as shown in Figure 3. The force *F_z_* has the effect on the lateral vibration of silicon wafer. Therefore, the effect of *F_z_* is only consider in the following calculation.

According to reference [27], the total normal cutting force for diamond wire sawing is:

(14) $$F_{N}=0.141\times \frac{v_{f}^{0.728}}{v_{s}^{0.656}}d^{1.017}L$$

where *v_f_* is the feed rate; *v_s_* is the wire speed; *d* is the diameter of the diamond wire; and *L* is the length of the silicon ingot.

Based on the relationship between forces in Figure 3, two forces can be obtained:

(14a) $$F_{n}\sin\alpha =F_{z}$$

(14b) $$F_{n}\cos\alpha =F_{y}$$

(15) $$\int_{0}^{b}\int_{0}^{\pi /2}F_{y}d\alpha dx=\frac{1}{2}F_{N}$$

By combining the above three equations, the force *F_z_* can be obtained as follows:

(16) $$F_{z}=\frac{1}{2b}F_{N}$$

Then in the x direction, the total transverse load *F_Z_* is:

(17) $$F_{Z}=\int_{0}^{b}\int_{0}^{\pi /2}\frac{1}{2b}F_{N}d\alpha dx=\frac{\pi }{4}F_{N}$$

Suppose that the sawing force is a harmonic force, the frequency is *ω_F_*, and the initial phase is *φ_F_*_:_

(18) $$F_{Z}=\frac{\pi }{4}F_{N}(\sin\omega_{F}t+\varphi_{F})$$

The distributed load *q* (*x*,*y*,*t*) can be expressed as:

(19) $$q(x,y,t)=F_{Z}/R$$

where *R* is the radius of diamond wire.

The transverse load *F_P_* on silicon wafer during sawing is:

(20) $$F_{P}=\int_{0}^{R}\int_{0}^{b}\frac{\pi }{4R}F_{N}(\sin\omega_{F}t+\varphi_{F})dxdy$$

(2) Forced vibration of silicon wafer

Cutting force applied to the silicon wafer induces wafer vibration, which is a forced vibration problem. The dynamic response of silicon wafer can be obtained based on the thin plate theory:

(21) $$w(x,y,t)=\sum_{m}^{\infty }\sum_{n}^{\infty }W_{mn}(x,y)Tt_{mn}(t)$$

where *W_mn_*, *Tt_mn_* are vibration mode obtained by Equation (7) and component of vibration mode respectively. Combining Equation (1), the forced vibration equation is:

(22) $$D\sum_{m}^{\infty }\sum_{n}^{\infty }W_{mn}\nabla^{2}\nabla^{2}T_{mn}+\rho h\sum_{m}^{\infty }\sum_{n}^{\infty }W_{mn}\overset{••}{T}t_{mn}=q(x,y,t)$$

On the basis of orthogonality between vibration modes, the following equation is satisfied:

(23) $$DW_{mn}\nabla^{2}\nabla^{2}=\rho h\omega_{mn}^{2}W_{mn}$$

Combining Equations (22) and (23), the following formula is obtained:

(24) $$\sum_{m}^{\infty }\sum_{n}^{\infty }\left[{\overset{••}{T}}_{mn}+\omega_{mn}^{2}Tt_{mn}\right]\rho hW_{mn}=q(x,y,t)$$

The above equation can be transformed another form about Ttmn by using orthogonality of vibration modes:

(25) $$\frac{d^{2}Tt_{mn}(t)}{dt^{2}}+\omega_{mn}^{2}Tt_{mn}(t)=\frac{F_{Pmn}(t)}{M_{mn}}$$

where *F_Pmn_* (*t*) is generalized force for (*m*,*n*) vibration mode; and *M_mn_* is generalized mass for (*m*,*n*) vibration mode. These two variables can be obtained from the Formulas (26) and (27):

(26) $$F_{Pmn}(t)=\iint_{s}q(x,y,t)W_{mn}(x,y)ds$$

(27) $$M_{mn}=\iint_{s}\rho hW_{mn}^{2}(x,y)ds$$

The solution for the Equation (25) can be obtained based on the vibration theory of single degree of freedom system:

(28) $$Tt_{mn}(t)=a_{mn}\cos\omega_{mn}t+b_{mn}\sin\omega_{mn}t+\frac{F_{p0}}{M_{mn}\omega_{mn}^{2}}Duhamel(t)$$

and these variables can be obtained by Formulas (29)–(32).

(29) $$a_{mn}=\frac{\iint_{s}\rho hw_{0}W_{mn}ds}{\iint_{s}\rho hW_{mn}^{2}ds}$$

(30) $$b_{mn}=\frac{\iint_{s}\rho h\overset{•}{w_{0}}W_{mn}ds}{\omega_{mn}\iint_{s}\rho hW_{mn}^{2}ds}$$

(31) $$F_{P}{}_{0}=\frac{\pi }{4}F_{N}$$

(32) $$Duhamel(t)=\frac{\omega_{mn}}{F_{P0}}\int_{0}^{t}F_{Pmn}(\tau )\sin\omega_{mn}(t-\tau )d\tau$$

Combing Equations (21) and (28), the vibration response of silicon wafer is:

(33) $$w(x,y,t)=\sum_{m}^{\infty }\sum_{n}^{\infty }W_{mn}(x,y)\left[a_{mn}\cos\omega_{mn}t+b_{mn}\sin\omega_{mn}t+\frac{F_{P0}}{M_{mn}\omega_{mn}^{2}}Duhamel(t)\right]$$

The Duhamel integral for the harmonic force is:

(34) $$Duhamel(t)=\frac{\omega_{mn}}{F_{P0}}\int_{0}^{t}F_{P}(\tau )\sin\omega_{mn}(t-\tau )d\tau =\frac{\frac{\omega_{mn}}{\omega_{F}}}{{\left(\frac{\omega_{mn}}{\omega_{F}}\right)}^{2}-{\left(2\pi \right)}^{2}}(\frac{\omega_{mn}}{\omega_{F}}\sin2\pi \omega_{F}t-2\pi \sin\omega_{mn}t)$$

Hence, the vibration response of silicon wafer can be rewritten as:

(35) $$w(x,y,t)=\sum_{m}^{\infty }\sum_{n}^{\infty }W_{mn}(x,y)\left[a_{mn}\cos\omega_{mn}t+b_{mn}\sin\omega_{mn}t+\frac{\frac{\pi }{4}F_{N}}{M_{mn}\omega_{mn}^{2}}\frac{\frac{\omega_{mn}}{\omega_{F}}}{{\left(\frac{\omega_{mn}}{\omega_{F}}\right)}^{2}-{\left(2\pi \right)}^{2}}(\frac{\omega_{mn}}{\omega_{F}}\sin2\pi \omega_{F}t-2\pi \sin\omega_{mn}t)\right]$$

It is assumed that the initial displacement *w*_0_ and velocity $\overset{•}{w_{0}}$ for silicon wafer are all zero. Therefore, the *a_mn_* and *b_mn_* are all zero based on the Equations (29) and (30). Thus, Equation (35) can be simplified as:

Hence, the vibration response of silicon wafer can be rewritten as:

(36) $$w(x,y,t)=\sum_{m}^{\infty }\sum_{n}^{\infty }W_{mn}(x,y)\left[\frac{\frac{\pi }{4}F_{N}}{M_{mn}\omega_{mn}^{2}}\frac{\frac{\omega_{mn}}{\omega_{F}}}{{\left(\frac{\omega_{mn}}{\omega_{F}}\right)}^{2}-{\left(2\pi \right)}^{2}}(\frac{\omega_{mn}}{\omega_{F}}\sin2\pi \omega_{F}t-2\pi \sin\omega_{mn}t)\right]$$

### 2.3. Stress in Silicon Wafer

The stress applied to silicon wafer is obtained based on the Kirchhoff plate theory. The stress *σ_z_* parallel to the neutral surface of silicon wafer is neglected as a secondary cause. Aside from that, the shear deformation induced by shear stress *τ_yz_* and *τ_xz_* are ignored as well. The other three stress *σ_x_*, *σ_y_* and *τ_xy_* distribute linearly along wafer thickness, which can be obtained by the following equation if the vibration of wafer has been known:

(37) $$\left\{\begin{array}{l} \sigma_{x}=-\frac{Eh}{1-\mu^{2}}\left(\frac{\partial^{2}w}{\partial x^{2}}+\mu \frac{\partial^{2}w}{\partial y^{2}}\right)\\ \sigma_{y}=-\frac{Eh}{1+\mu }\left(\frac{\partial^{2}w}{\partial y^{2}}+\mu \frac{\partial^{2}w}{\partial x^{2}}\right)\\ \tau_{xy}=-\frac{Eh}{1+\mu^{2}}\frac{\partial^{2}w}{\partial x\partial y} \end{array}\right.$$

The principal stress for an arbitrary point in the wafer is:

(38) $$\sigma =\frac{\sigma_{x}+\sigma_{y}}{2}+\sqrt{{\left(\frac{\sigma_{x}-\sigma_{y}}{2}\right)}^{2}+\tau_{xy}^{2}}$$

Thus, the maximum principal stress for an arbitrary point in the silicon wafer is obtained, which is used to calculate the breakage ratio of wafers.

### 2.4. Breakage Ratio for Wafer during Wire Sawing

Stress and machining damage are the major factors influencing wafer breakage. Machining damage such as micro crack, residual stress, dislocation and slip are inevitable during diamond wire sawing, which have different effects on the wafer strength. Lots of researchers have been studied the influence of machining damage on sawn wafer strength. Their experimental results are used for as sawn silicon wafer in this part. The principal stress for an arbitrary point in the silicon wafer is compared to obtain the maximum principal stress which is used to calculate the wafer breakage ratio.

Based on the probabilistic fracture mechanics, the break probability for wafers is:

(39) $$P(\sigma )=1-\exp\left[-\int_{A}{\left(\frac{\sigma_{\max}}{\sigma_{0}}\right)}^{m_{A}}dA\right]$$

where σ_max_ is the maximum applied stress in silicon wafer; σ_0_ is scale parameter of Weibull distribution; *m_A_* is the shape parameter of Weibull distribution. From the reference [28], these two Weibull parameters are taken 151.3 Mpa and 3.5.

## 3. Results and Discussion

The breakage ratio of sawn silicon wafers during diamond wire sawing can be obtained by using the established model. The used parameter values are shown in Table 1.

Each order nature frequency of silicon wafer is solved by Equations (8)–(11) for different cutting depth. After that, vibration shape function is obtained using Equation (7) through solving the four unknown parameters *I_m_*, *J_m_*, *K_n_* and *L_n_*. Then the vibration response of wafer, stress and breakage ratio can be solved gradually by using the established theoretical model.

### 3.1. Free Vibration

The first fourth modes of nature frequency are calculated for sawn wafer when cutting depth is 156 mm, and the FEM simulation is adopted to get the natural frequency in order to verify the correctness of the established model. The results obtained by these two methods are shown in Table 2. It can be seen that, the results are comparable, which indicates the correctness of this established model.

**Figure 4:**
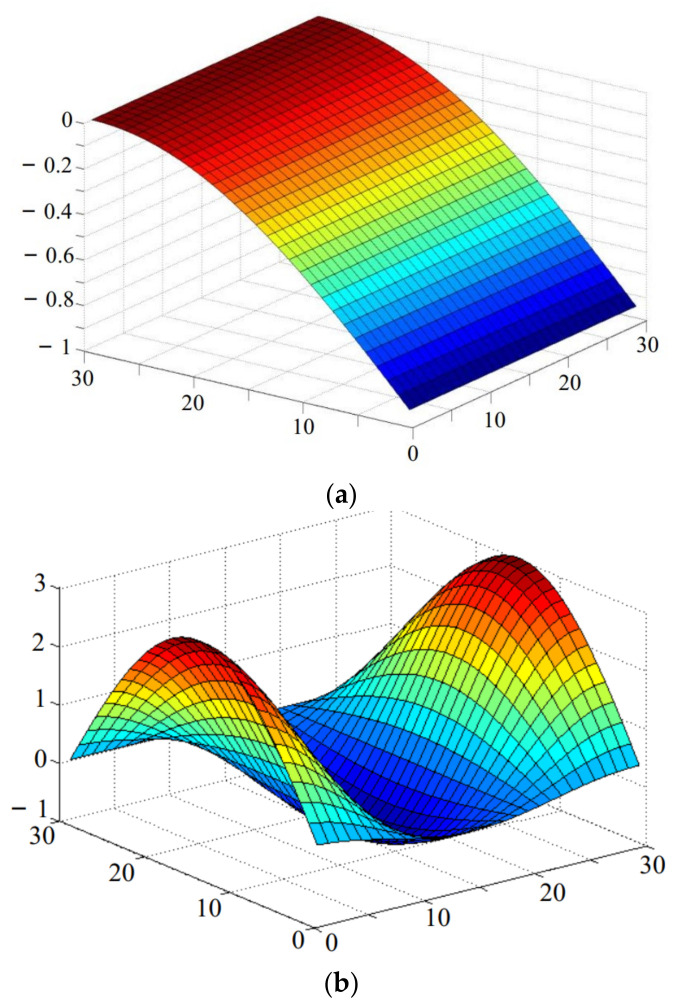

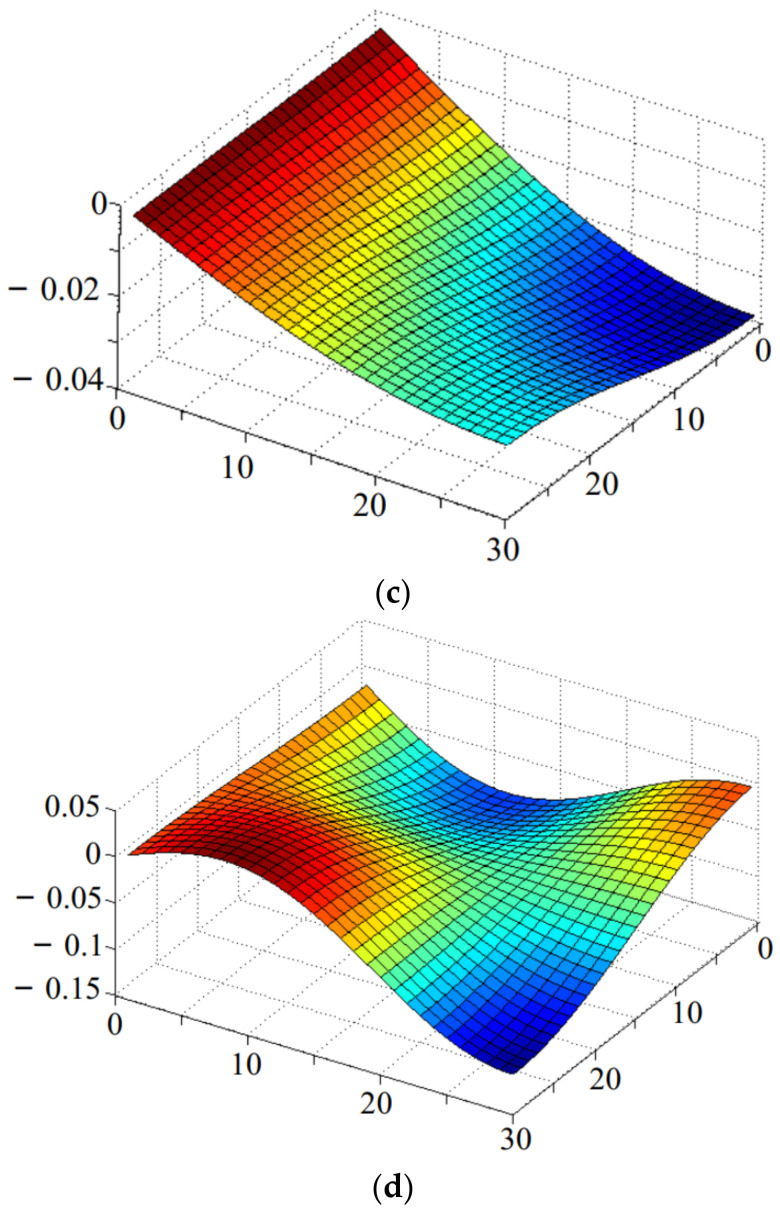
Vibration mode of free vibration for sawn silicon wafer. (**a**) First order of symmetrical mode. (**b**) Second order of symmetrical mode. (**c**) First order of anti-symmetrical. (**d**) Second order of anti-symmetrical.

The symmetrical mode and anti-symmetrical mode vibration are obtained by setting *I_m_* = −*J_m_* and *I_m_* = *J_m_* respectively, as shown in Figure 4.

### 3.2. Forced Vibration and Stress in Silicon Wafer

**Figure 5:**
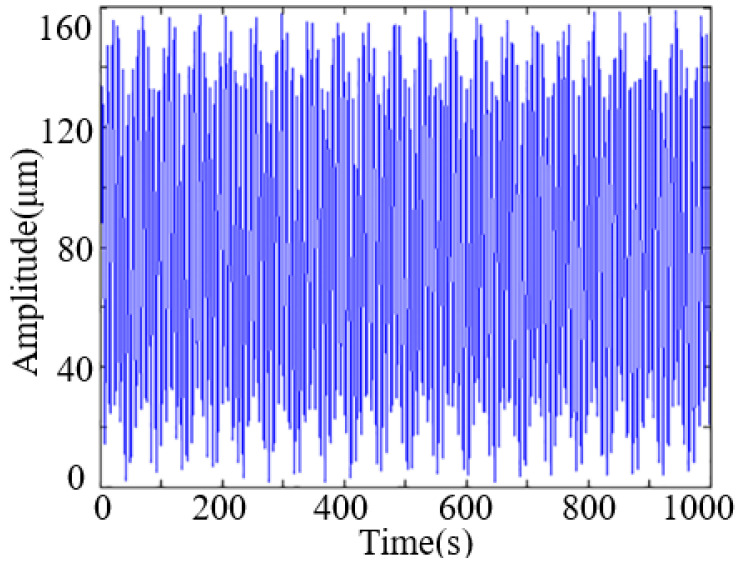
Vibration state of point (*b/*2, *a*) when cutting depth is 156 mm.

The amplitude of the wafer is the superposition of each vibration order. Since the natural frequency of the silicon wafer increases with the increase of the order, the amplitude caused by the vibration of the higher order is very small. Therefore, the transverse vibration of silicon wafer is mainly generated by the superposition of lower order vibration. The maximum amplitude is recorded with the changes of cutting depth. The vibration sate of the point (*b/*2, *a*) is shown in Figure 5 when the cutting depth is 156 mm. It can be seen the maximum vibration amplitude is about 160 μm when wire speed is 2 m/s and feed rate is 6 μm/s. The excitation frequency is 50 Hz, thus the amplitude of force vibration at the edge point (*b*/2, *a*) of the silicon wafer, so the amplitude variation period at this point is also 50 Hz.

**Figure 6:**
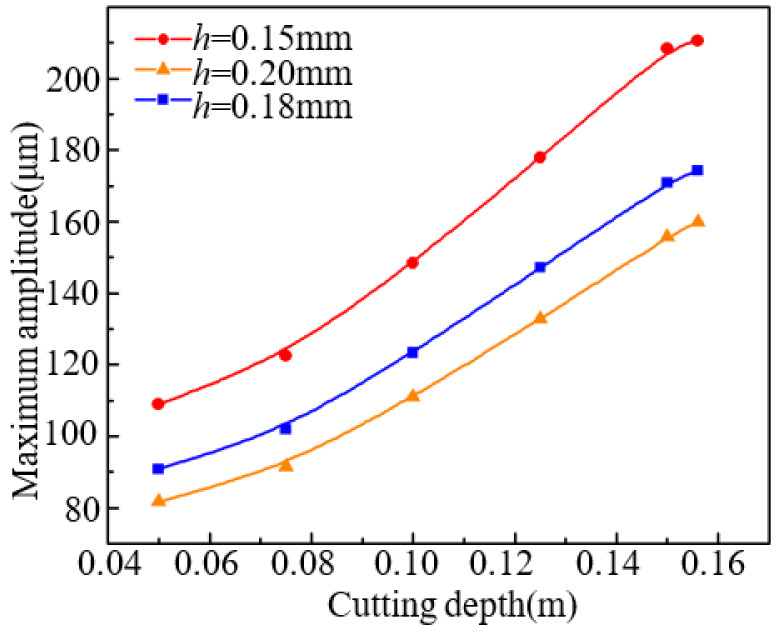
Changes of maximum amplitude for three kinds of as-sawn wafers.

Different cutting depths lead to the maximum vibration amplitude is different, the results are shown in Figure 6. The maximum vibration amplitude becomes larger with the increase of cutting depth. In addition, the maximum vibration amplitudes for different thickness silicon wafers are compared. The variation tendency of maximum amplitude for these three kinds of sawn silicon wafer is similar. The thinner of silicon wafer, the larger vibration amplitude.

**Figure 7:**
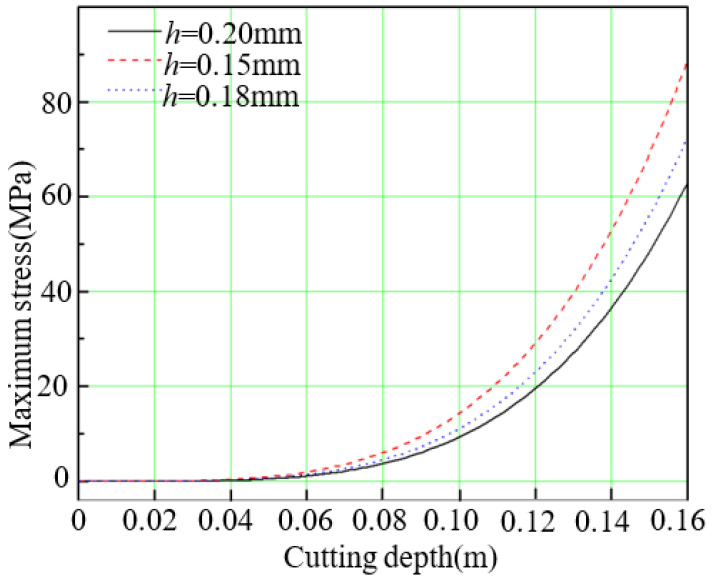
Changes of maximum stress with cutting depth for three kinds of wafer.

Larger vibration amplitude leads to larger maximum stress for silicon wafer as shown in Figure 7. Because of the distance between free side and fixed side is larger with increase of cutting depth, the maximum stress increase gradually as well. The variation trend of maximum stress is accord with the changes of maximum vibration amplitude. At the initial stage of diamond wire sawing, the maximum stress changes gently. While at the end of diamond wire sawing, the maximum stress increases sharply. This kind of change of maximum stress accords with the changes of maximum amplitude. Moreover, it indicates that the end stage of diamond wire sawing is difficult to handle because of larger maximum stress. If the adhesive strength between wafer and basal plate is not larger enough, the wafer may drop out and generate waste wafer.

### 3.3. Breakage Ratio for As-Sawn Silicon Wafer

**Figure 8:**
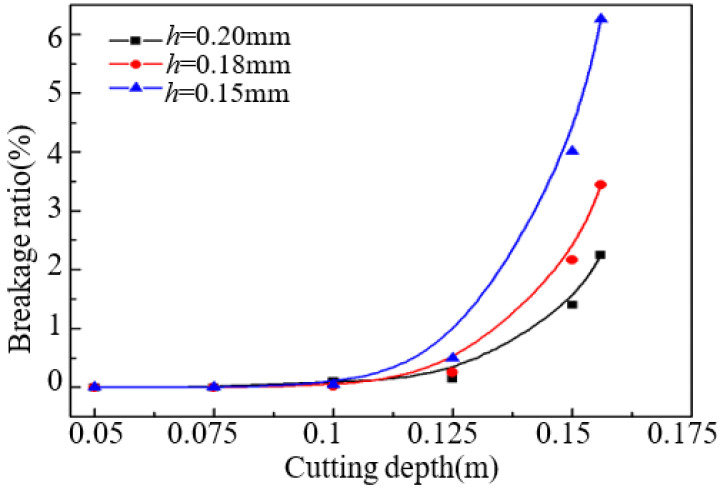
Changes of breakage ratio with cutting depth for three kinds of wafer.

Take the same cutting parameters as above, the results of breakage ratio with the increase of cutting depth for silicon wafers with different thickness are shown in Figure 8. It can be seen that, the breakage ratio is very small and changes relatively little when the cutting depth is less than 0.1 m. While the breakage ratio increases sharply when the cutting depth is larger than 0.125 m for these three kinds of silicon wafer. The trend of wafer breakage ratio is accord with the changes of vibration amplitude and maximum stress.

During calculate the breakage ratio for silicon wafer, the same machining damage on the three kind of silicon wafer is assumed. It means the Weibull parameters for Weibull strength distribution are same. Thus, the breakage ratio is concerned with wafer surface area and stress. From the analysis previous, the maximum stress and surface area become larger with the increase of cutting depth. This leads to the increase of wafer breakage ratio with increase of cutting depth. Furthermore, thinner silicon wafer has larger breakage ratio because of larger maximum stress. When the cutting stage is completed, the breakage ratio for 0.15 mm wafer is about 6%, while that for 0.2 mm wafer is about 2%.

## 4. Conclusions

In this paper, a mathematical model for calculating breakage ratio of silicon wafer is established based on the fracture mechanism. The maximum stress and breakage ratio for as-sawn silicon wafer are studied. This paper first study the breakage ratio of silicon wafers during diamond wire sawing. The results can be used to optimize processing parameters for diamond wire saw in order to thinning wafer thickness and increasing yield. The main conclusions of this paper are as follows:

(1) The forced vibration model of the wafer during the sawing process was established, and the steady-state solution of the forced vibration of the wafers was obtained. The first five natural frequencies of the wafers were calculated using the finite element and the model of this paper respectively. The maximum relative error of the two methods was 3.43%, indicating the accuracy of the model in this paper.
(2) The maximum amplitude of silicon wafer with the size of 156 mm × 156 mm × 0.2 mm was 160 μm during the diamond sawing process. The amplitude, maximum stress and breakage ratio of wafers increased with the increase of the cutting depth, and the smaller the wafer thickness, the larger the increase.
(3) In the sawing stage, the breakage ratio of the 156 mm × 156 mm section with a thickness of 0.15 mm silicon wafer is 6%.

## Figures and Tables

**Table 1:** Parameter values used in calculation.

Parameter	Value	Unit
Density *ρ*	2330	kg/m^3^
Poisson ratio *μ*	0.22	
Sawn wafer thickness *h*	0.2	mm
Modulus of elasticity *E*	165	Gpa
Wafer length *a*	156	mm
Wafer width *b*/*L*	156	mm
shape parameter *m_A_*	3.5	
scale parameter σ_0_	151.3	Mpa
Wire diameter *d*	100	μm
Wire speed *v_s_*	2	m/s
Feed rate *v_f_*	6	μm/s
Excitation frequency *ω_F_*	50	Hz

**Table 2:** Natural frequency obtained by theoretical model and FE.

Computational Method	First Order Natural Frequency	Second Order	Third Order	Fourth Order
Theoretical model	69.32	417.9	548.4	1084.7
FEM	67	405.6	532.5	1078.6
